# Correction to ‘DNA-protein Interactions at the Telomeric Repeats of *Schizosaccharomyces Pombe’*

**DOI:** 10.1093/nar/gkaf1181

**Published:** 2025-10-30

This is a correction to: Margaret Duffy, Alistair Chambers, DNA-protein Interactions at the Telomeric Repeats of *Schizosaccharomyces Pombe, Nucleic Acids Research*, Volume 24, Issue 8, 1 April 1996, Pages 1412–1419, https://doi.org/10.1093/nar/24.8.1412

The figures published in the HTML version of this manuscript are incorrect. The correct figures are shown in the PDF version.

These details have been corrected only in this correction notice to preserve the published version of record.

